# Post-Translational Mechanisms of Plant Circadian Regulation

**DOI:** 10.3390/genes12030325

**Published:** 2021-02-24

**Authors:** Jiapei Yan, Yeon Jeong Kim, David E. Somers

**Affiliations:** Department of Molecular Genetics, The Ohio State University; Columbus, OH 43210, USA; yan.424@osu.edu (J.Y.); kim.6352@osu.edu (Y.J.K.)

**Keywords:** phosphorylation, ubiquitination, SUMOylation, methylation, nucleocytoplasmic partitioning, O-glycosylation, phosphatidic acid, intercellular/interorgan coupling, circadian clock, Arabidopsis

## Abstract

The molecular components of the circadian system possess the interesting feature of acting together to create a self-sustaining oscillator, while at the same time acting individually, and in complexes, to confer phase-specific circadian control over a wide range of physiological and developmental outputs. This means that many circadian oscillator proteins are simultaneously also part of the circadian output pathway. Most studies have focused on transcriptional control of circadian rhythms, but work in plants and metazoans has shown the importance of post-transcriptional and post-translational processes within the circadian system. Here we highlight recent work describing post-translational mechanisms that impact both the function of the oscillator and the clock-controlled outputs.

## 1. Introduction

In circadian biology, efforts in understanding the regulatory mechanism of the circadian clock have long been focused on transcriptional studies, leading to the well-established transcriptional translational feedback loops (TTFL) model [1,2,3]. However, time-course transcriptome and proteome comparisons have found that changes in transcript abundance often do not correspond to protein abundance changes. Additionally, significant differences between peak mRNA and protein expression [4,5] indicate that proteins often do not simply follow transcript abundance patterns, suggesting that protein turnover and post-translational modifications (PTMs) play prominent roles in clock regulation. We consider and review recent progress in understanding post-translational mechanisms in the control of the plant circadian clock.

## 2. Post-Translational Mechanisms: Protein Modifications

### 2.1. Ubiquitination

Expression of most circadian clock genes oscillate robustly over the course of a day during which positive and negative regulators cooperatively and antagonistically modulate the transcription of their oscillation. The regulators are often proteins with a short half-life [6,7,8,9,10,11,12,13,14] that enable phase-specific effects on the respective downstream genes. Such limited protein stability is often important for circadian oscillations, where no long-lasting regulation predominates.

Numerous studies have shown that ubiquitination, occupies a prominent place in the turnover of circadian clock proteins [15]. Ubiquitination is a proteolytic pathway whereby targets are polyubiquitinated by covalent attachments of ubiquitin chains of 76 amino acid monomers which flag the substrate for cleavage into constituent amino acids, mediated by the 26S proteasome complex [16,17].

In Arabidopsis the first successful studies that identified turnover factors involved in the circadian clock implicated components of the CUL1-based E3 ligase or SCF (SKP1-like Cul1 F-box) [18], in which the substrate adaptor is the ZEITLUPE (ZTL) three-member family of F-box proteins [19,20,21]. ZTL, FLAVIN-BINDING, KELCH REPEAT, F-BOX 1 (FKF1), and LOV KELCH PROTEIN 2 (LKP2) all possess a single Light, Oxygen, or Voltage (LOV) photosensory domain at the N-terminus which covalently binds flavin mononucleotide and is responsive to blue light, similar to the blue-light photoreceptor phototropin [22,23,24]. C-terminal to the LOV domain are F-box and Kelch repeat domains.

Dedicated studies showed that the ZTL protein family indeed associates with SCF complex members [25,26,27,28]. Later work first identified TIMING OF CAB1 (TOC1) and PSEUDO-REPSONSE REGULATOR 5 (PRR5) as the primary ubiquitination targets of ZTL, and more recently the transcription factor CCA1 HIKING EXPEDITION (CHE) [8,9,13,14,27] with the interactions mediated by the LOV domain [9,14,27,28]. Other PRR members, PRR9, PRR7, and PRR3 do not interact with ZTL family members [13,28,29], but PRR3-TOC1 interaction alters the access of ZTL to TOC1 in the cytoplasm [29,30].

ZTL is itself a substrate for ubiquitination, and this PTM is likely responsible for the rhythmic expression of ZTL since *ZTL* transcripts are expressed constitutively [7]. Robust ZTL oscillations require GIGANTEA (GI) interaction with the LOV domain, which co-stabilizes both proteins under blue light [31]. This binding consequently results in peak ZTL abundance in the evening, in phase with maximum GI expression. 

The GI-mediated ZTL stabilization also requires heat shock protein 90 (HSP90), which interacts with and prevents ZTL from aggregation. HSP90 depletion and treatment with geldanamycin, a specific inhibitor of HSP90, decreases ZTL abundance and lengthens the circadian period [32]. Subsequent work showed GI and HSP90 cooperatively act in the maturation and stabilization of ZTL [33]. Both in vitro and in vivo experiments showed that GI functions as a co-chaperone with HSP90, with GI/ZTL/HSP90 trimeric complexes implicated and detected through gel filtration and co-immunoprecipitation experiments. The interaction of GI with HSP90, and the oscillation of GI protein arising from the circadian regulation of its transcription, confers a circadian rhythmicity to the fundamental chaperone activity of HSP90 when coupled to the co-chaperone GI. Given the wide range of putative and proven GI interactors [34], the GI-HSP90 relationship is a likely mechanism for proteome-wide circadian control of proteostasis [35].

An additional role of GI in the ZTL complex includes recruitment of the deubiquitinases UBP12 and UBP13, which catalyze cleavage of Ub chains from ubiquitin conjugates [27,34]. GI acts as a linker between the UBPs and ZTL to form a ternary complex [36]. The *ubp12* and *ubp13* mutations reduce the abundances of GI and ZTL suggesting that excessively ubiquitinated forms of both proteins leads to their degradation. Surprisingly, peak TOC1 levels are also reduced, resulting in a reduction in TOC1 amplitude and a short circadian period, similar to the effect of a *gi* loss of function mutation. Lee and coworkers [36] highlighted how a balance between ubiquitination and de-ubiquitination helps maintain circadian proteostasis. Similarly, another class of deubiquitylating enzymes, *UBIQUITIN CARBOXYL-TERMINAL HYDROLASES* (*UCH*) also influence clock function. The triple mutation of *uch1, uch2*, and *uch3* markedly lengthens the circadian period at high temperature, although the substrates have not been identified [37].

Other ZTL family members, FKF1 and LKP2, might be under the GI-mediated regulation as well [27,34,38]. However, the extent of their role in circadian control is not as strong as ZTL [14]. A recent study using a decoy system showed that circadian period is strongly altered by overexpression of an F-box-deleted ZTL and LKP2, but only slightly by FKF1 [27]. This result confirms that the circadian regulation of ZTL and LKP2 rely on their substrate ubiquitination activities, as the decoy proteins still bind the targets but do not allow their proteolysis. Additionally, the same decoy strategy suggests the possibility of more F-box proteins involved in circadian regulation, and two U-box genes, *PLANT U-BOX 59* (*MAC3A*) and *PLANT U-BOX 60* (*MAC3B*) were found necessary for the normal splicing of *PRR9* [39].

While a ZTL-GI blue-light mediated interaction co-stabilizes both proteins in the cytosol, dark-induced degradation of GI is accomplished by the CONSTITUTIVE PHOTOMORPHOGENIC 1/SUPPRESSOR OF PHYTOCHROME A-105 1 (COP1/SPA1) complex, which was first characterized as a negative regulator of photomorphogenesis [40,41]. COP1 is an E3 ligase, modulated by SPA1 [42], and in this model EARLY FLOWERING3 (ELF3) acts as a substrate adaptor between COP1 and GI to facilitate destabilization of GI in the nucleus in the dark, promoting the turnover of both GI and ELF3 [41]. Some evidence also suggests that COP1-dependent turnover of GI is enhanced at low temperatures due to increased COP1 stability [40].

COP1-mediated degradation of ELF3 is also facilitated by the B-BOX protein BBX19 [43]. This transcription factor interacts with both COP1 and ELF3 to recruit ELF3 to the COP1 complex in the nucleus, acting as a linker between COP1 and ELF3 similar to the role ELF3 plays between COP1 and GI. BBX18 and BBX23 may function similarly in COP1-mediated regulation of ELF3 during thermomorphogenesis [44].

Central clock components CIRCADIAN CLOCK ASSOCIATED 1 (CCA1) and LATE ELONGATED HYPOCOTYL (LHY) interact with DE-ETIOLATED 1 (DET1) [45] which is part of a COP10-DET1-DDB1-CUL4 complex [46]. This interaction with DET1 occurs at the chromatin, with DET1 anchored to the DNA via LHY/CCA1 and acting as a co-repressor to regulate LHY/CCA1 dependent gene expression [45]. Evidence of effects of the DET1-LHY interaction on LHY protein abundance comes from enhanced protein stability of LHY through DET1-dependent perturbation of the binding of the SINAT5 E3 ubiquitin ligase to LHY in vitro [47]. This is consistent with a report of more rapid in vitro degradation of LHY in *det1* mutants [48], but further in vivo work showing *sinat5* mutant effects on LHY/CCA1 levels or circadian period have not been published.

Collectively, these studies illustrate the variety of mechanisms that control protein abundance in the plant circadian system. Each is ultimately anchored in ubiquitin-based and proteosome-mediated degradation, but the factors initiating the process are still unknown for most clock proteins [6,7,49,50,51,52]. Additionally, initiation of ubiquitin-based degradation can also be influenced by subcellular positioning and other post-translational modifications, as noted below.

### 2.2. Phosphorylation

Phosphorylation of key clock proteins is an important post-translational modification for sustaining the circadian system in *Neurospora*, *Drosophila*, and mammals [53,54,55], and is also involved in the clock output pathways [56]. CASEIN KINASES (CK) are conserved across species to phosphorylate many of the essential clock proteins, such as BMAL1 and PERIOD2 (PER2) in mammals and PERIOD (PER) and TIMELESS (TIM) in *Drosophila*, and FREQUENCY in *Neuropsora* [57,58,59,60,61,62,63].

The first evidence of the involvement of CKs in regulating the plant clock came from a yeast two-hybrid screen in which CKB3, the β subunit of CK2, was identified as an interacting partner of a key component of *Arabidopsis* central oscillator, CCA1 [64]. Further studies showed that CK2 phosphorylates CCA1 and its closely related homolog, LHY, in vitro, and phosphorylation of CCA1 is required for its DNA binding to the LIGHT-HARVESTING CHLOROPHYLL A/B1*3 (LHCB1*3) promoter [64,65]. Consistent with this, the *cka1a2a3* triple mutant results in reduced CCA1 phosphorylation, circadian period lengthening and a decreased photoperiodic flowering response [66]. Ectopic expression of *CKB3* or *CKB4* results in elevated CK2 activity and shortens the circadian period similar to the effects of *cca1* and *lhy* mutants [52,65]. Conversely, knockdown of the CKB3 gene family lengthens the circadian period, similar to the *cka1a2a3* triple mutant [67].

**Figure 1:**
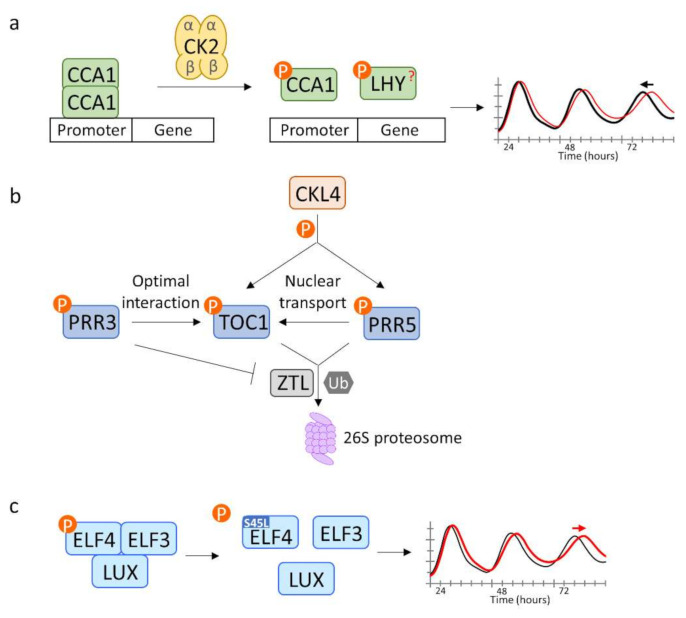
Phosphorylation of clock proteins modulates their function in regulating circadian rhythms. (**a**) CK2 phosphorylates CCA1 and interferes with its binding to target promoters, leading to reduced transcriptional activity of CCA1 and a shortened circadian period (LHY? indicates CK2 phosphorylates LHY in vitro, but in vivo evidence has not yet been reported). (**b**) CKL4 phosphorylates TOC1 and PRR5. Phosphorylation of TOC1 and PRR5 enhances their interaction with ZTL and subsequent proteasomal degradation. Phosphorylation also stabilizes TOC1 by PRR5-mediated nuclear sequestration and by competitive interaction with PRR3 which protects TOC1 from ZTL-targeted proteasomal degradation. It is not known whether CKL4-mediated phosphorylation is responsible for the interaction and/or transport features of TOC1 and PRR5. (**c**) Mutation of the ELF4 phosphorylation site diminishes interaction with ELF3 and lengthens circadian period.

Later analysis of the role of CKB4 showed that CK2 does not alter protein accumulation or subcellular localization of CCA1, but interferes with the transcriptional activity of CCA1, with dephosphorylated CCA1 protein preferentially bound to the promoters of its target clock genes [68] (Figure 1). Stronger promoter binding of dephosphorylated CCA1 to key clock genes is consistent with the long period of the *cka1a2a3* mutant and CKB3 gene family knockdown. High temperature enhances both CCA1 binding and CK2 phosphorylation (which should reduce binding). These opposing outcomes by CK2 and CCA1 are proposed to balance and maintain a stable period across a physiological range of temperatures, suggesting a molecular mechanism underlying temperature compensation of the *Arabidopsis* clock [68]. However, it is possible that CKs are involved in the phosphorylation of other clock proteins as well.

Another core group of oscillator genes is comprised of five PSEUDO-RESPONSE REGULATOR (PRR) proteins, including TOC1, PRR3, PRR5, PRR7 and PRR9, all of which are phosphorylated in a time-of-day dependent manner [29]. The phosphorylation of both TOC1 and PRR3 is necessary for their optimal interaction [29]. As PRR3 and ZTL interact with TOC1 through the same N-terminal region, PRR3 perturbs TOC1 interaction with ZTL and protects TOC1 from proteasome-dependent degradation [29,30]. At the same time, phosphorylation of PRRs likely makes them more susceptible to degradation, as the affinity of PRR5 and TOC1 with ZTL is enhanced by phosphorylation [29]. PRR5-TOC1 interaction promotes TOC1 phosphorylation and facilitates TOC1 transport into the nucleus, which may shield TOC1 from cytoplasmic ZTL-dependent degradation [8]. These findings suggest a complex interplay between phosphorylation and stability regulating PRR interactions, location and abundance, thereby modulating their clock functions (Figure 1).

To better elucidate phosphorylation events tied to circadian system, a quantitative phosphoproteomic analysis of Arabidopsis identified extensive cyclic changes in the phosphorylation state of a wide range of physiological, metabolic, and signaling components [69]. Phosphorylation of Ser 45 of EARLY FLOWERING 4 (ELF4) oscillates in constant light (LL) while ELF4 protein does not. The S45L variant has a slightly longer period, especially at high temperatures, and some alterations in gene expression [69,70]. This mutation also diminishes interaction with ELF3, a key partner in a tripartite evening complex (EC) required for circadian cycling [69,71,72]. Recent work indicating that the temperature sensitivity of ELF3 is modulated by ELF4 [73] aligns with a compromised temperature compensation resulting from weaker binding between ELF4^S45L^ and ELF3 [69] (Figure 1). In addition to ELF4, this circadian phosphoproteome study identified a number of factors exhibiting rhythmic oscillations in their phosphorylation state, yielding potential candidates for further investigation of the role of phosphorylation-dependent regulation of the clock [69].

Apart from the casein kinase/CCA1 relationship, the kinases responsible for clock protein phosphorylation are largely unknown. Transcriptome analysis revealed that expression of a large number of protein kinases and phosphatases is clock-regulated [3,5,74,75]. However, genome duplication, genetic redundancy and null mutant lethality has made it difficult to associate specific kinases with their targets. A recent screen for pharmacologically-active compounds identified two small molecules, PHA767491 and 3,4-dibromo-7-azaindole (B-AZ), which significantly lengthen circadian period in a dose-dependent manner in plants [76,77]. PHA767491 was originally found as cell division cycle 7 (CDC7) and CDK9 inhibitor in mammals [78]. An affinity-based proteomic approach identified kinases from the CK1 family (CKL) in Arabidopsis, which is comprised of 13 members, as direct targets of PHA767491 [77]. Reduced function of CKLs results in circadian period lengthening similar to PHA767491 treatment. Further in vitro kinase assays and in vivo band shift assays showed CKL4 can phosphorylate PRR5 and TOC1, and is inhibited by PHA767491. PHA767491 induces overaccumulation of PRR5 and TOC1 accompanied with decreased expression of PRR5 and TOC1 target genes [77]. A *prr5 toc1* double mutant was hyposensitive to PHA767491-induced circadian period lengthening, indicating PHA767491 and CKL modulate the circadian period through PRR5 and TOC1 [77]. B-AZ, though different in structure from PHA767491, was also found to lengthen the circadian period, inhibit CKL4 activity, and promote accumulation of PRR5 and TOC1. A docking study and molecular dynamics simulation suggested that PHA767491 and B-AZ interact with the ATP binding pocket of human CK1δ by forming a hydrogen bond with Leu 85, which is a highly conserved residue among human CK1δ and Arabidopsis CKLs [76]. Whether CKL4 is the sole kinase acting on these two PRRs, and which sites are phosphorylated, is still unknown (Figure 1).

Global transcriptome and proteome comparison during the light-dark transition between wild type and clock mutants identified 60 protein kinases which have significant transcript level changes in clock mutants, including sucrose non-fermenting-related (SnRK), calcineurin B-like (CBL) interacting kinases (CIPKs), uncharacterized leucine-rich repeat (LRR) or LRR-like and cysteine-rich receptor-like (RLK) protein kinases [5]. A recent MS-based proteomics and phosphoproteomics study over a circadian time course reported that rhythmic protein phosphorylation is more wide-spread than rhythmic protein abundance, and that most rhythmic phosphopeptides peak at subjective dawn [79]. Kinase prediction and enrichment analysis of the subjective-dawn phased phosphopeptides indicated that the CDPK-SnRK superfamily of kinases in plants is most consistently enriched in the different datasets. SnRK1 has been suggested as a strong candidate protein kinase for this phospho-dawn process based on its relevance to circadian timing and profound effects on clock output pathways [79]. This is consistent with a report that KIN10, the catalytic subunit of SnRK1, is essential in linking sugar signaling to circadian entrainment through the binding of the transcription factor bZIP63 to the *PRR7* promoter [80].

A large number of the rhythmic phosphosites reported in the Krahmer et al. report have not been previously characterized [79]. Additional studies involving systematic quantification of phosphoproteomes using different tissues under diurnal conditions have been published recently [81,82]. Mining these works may provide insights into the connection between rhythmic phosphorylation changes and developmental/metabolic processes, and suggest kinases that are involved in these phosphorylation events.

### 2.3. O-Glycosylation

Protein *O*-GlcNAcylation, mediated by O-linked N-acetylglucosamine transferases (OGTs), can set circadian clock speed through the regulation of nuclear entry, and by contributing to the stability of core clock proteins in *Drosophila* and mice [83,84]. SPINDLY (SPY) and SECRET AGENT (SEC) were both predicted to encode OGTs in higher plants, as they share similarity with animal OGTs in containing an N-terminal tetratricopeptide repeat (TPR) domain and a C-terminal putative OGT catalytic domain [85]. Mass spectrometry (MS) analyses showed that SEC does *O*-GlcNAcylate the DELLA protein RGA in *Arabidopsis*. However, SPY acts as an *O*-fucosyltransferase (POFUT), which modifies RGA by attaching monofucose to specific serine and threonine residues [85,86].

Wang et al. recently reported that *spy,* but not *sec,* mutants show a significantly lengthened circadian period in both Col-0 and L*er* backgrounds [87]. However, unlike an earlier report showing that SPY physically interacts with GI in yeast and in vitro [88], affinity purification followed by mass spectrometry (AP-MS) identified PRR5, not GI, as a target of SPY, and further assays confirmed SPY *O*-fucosylates PRR5 *in planta* [87]. SPY *O*-fucosylation of PRR5 controls period through enhancing PRR5 proteolysis and alleviating PRR5-repressed target gene expression [87]. As SPY *O*-fucosylates serine and threonine residues [85], which alternatively can be phosphorylated, it is tempting to speculate that *O*-fucosylation may regulate clock protein activity and stability by affecting PRR5 phosphorylation status. Crosstalk between *O*-GlcNAcylation and phosphorylation has been well documented [89], and recent work in the vernalization field highlights the dynamic interaction between phosphorylation and *O*-glycosylation in the regulation of gene expression in plants [90,91]. Further studies focusing on identification of the *O*-fucosylated and phosphorylated residues of PRR5 and examination of other clock proteins that might be modified by *O*-glycosylation are needed for a better understanding of the role of *O*-glycosylation in modulating circadian period.

### 2.4. SUMOylation

SUMOylation is the post-translational modification of proteins which covalently conjugates Small Ubiquitin-related Modifier (SUMO; ~100 amino acids) to a lysine residue in the target substrate [92]. SUMOylation is highly dynamic and reversible and post-translational regulation by SUMOylation plays essential roles in developmental processes and stress responses in plants [93]. SUMOylation regulates protein activity by inducing subcellular redistribution, modulating protein–protein interactions, competing with other post-translational modifications, promoting protein conformational changes or target protein for ubiquitination and subsequent degradation by the proteasome [92,94].

Within circadian systems SUMOylation of BMAL1, an essential transcription factor in the mammalian clock, oscillates in a circadian-dependent manner and parallels BMAL1 activation. Loss of SUMOylation in BMAL1 results in altered period [95]. In plants, the double mutant of the *Arabidopsis* SUMO proteases OVERLY TOLERANT TO SALT1 and 2 (*ots1 ots2*), exhibits an increased level of overall SUMOylation and a markedly lengthened circadian period [96]. On the other hand, a mutant of the SUMO ligase SIZ1 shows overall reduced levels of SUMOylation and a short circadian period, supporting the notion that SUMOylation can modulate clock function in plants [96].

Analyses of circadian rhythm in *ots1 ots2* and *siz1* mutants at different temperatures showed that the clock in *siz1* is undercompensated at higher temperatures, whereas the clock in *ots1 ots2* is undercompensated at the lower temperatures, suggesting the level of SUMOylation contributes to temperature compensation of clock [96]. Subsequent work showed CCA1 SUMOylation in vivo oscillates in similar proportion to the abundance of the protein [97]. Neither the localization nor the stability of CCA1 is significantly affected by its SUMOylation state. However binding of CCA1 to the evening element within the *PRR9* promoter was significantly reduced in the *ots1 ots2* mutant, indicating SUMOylation suppresses the binding activity of CCA1 to target genes [97].

Interestingly, a recent analyses of the proteome and phosphoproteome of SUMOylation mutants in Arabidopsis found a high abundance of predicted SUMO attachment sites in phosphoproteins [98]. This is notable in that CCA1 phosphorylation reduces promoter binding [68] while SUMOylation also suppresses CCA1 binding activity, encouraging further investigation into the relationship between phosphorylation and SUMOylation in clock proteins.

### 2.5. Protein Methylation

Protein arginine methylation is one of the most abundant post-translational modifications in eukaryotes and plays an essential role in mediating diverse cellular processes, such as transcriptional regulation and RNA processing [99,100,101]. A type II protein arginine methyltransferase, PRMT5, is well-conserved among yeast, animals and higher plants, exhibiting dual nuclear-cytoplasmic localization and catalyzing symmetric dimethylation of arginine residues in histone and non-histone proteins [99,102,103,104]. PRMT5 can methylate components of the transcription complex, such as SPT5, altering its interaction with RNA polymerase II and potentially affecting global transcription rates [105].

PRMT5-mediated histone methylation often functions in repressing target gene expression, and its absence results in pleiotropic developmental and flowering defects in plants [106,107,108]. Another role for PRMT5 is methylation of Sm spliceosomal proteins that are essential RNA processing factors, with *prmt5* mutants showing broad RNA splicing defects in many genes involved in multiple biological processes in plants [109], including the circadian clock.

Two forward genetic screens in *Arabidopsis* each independently isolated long period mutants in *PRMT5*, providing first-time evidence for a connection between protein arginine methylation and the circadian system [110,111]. Transcript abundance of PRMT5 oscillates and responds to both light and temperature cues, suggesting that PRMT5 participates in a feedback loop within the *Arabidopsis* clock [110,111]. Genome-wide transcriptome abundance and pre-mRNA splicing analyses uncovered a significantly altered gene expression profile, increased intron retention, and enrichment in alternative 5′ splice sites in *prmt5* mutants, which suggested improper splicing [111]. An alternatively spliced isoform of PRR9 that retains intron 3 overaccumulates in *prmt5*, whereas the isoform encoding the full-length protein is significantly reduced [111].

PRMT5 also affects *PRR7* expression but not its splicing, although genetic analysis indicates both *PRR7* and *PRR9* are required to account for PRMT5 effects on the clock [111,112]. In addition to PRR9, other clock-associated genes were also reported as potential targets of PRMT5, for instance, the amplitude of *GI* increases in *prmt5* mutant [110]. However, the functional significance of this observation remains elusive and regulatory mechanisms other than alternative splicing cannot be excluded due to the broad role of PRMT5 in protein arginine methylation. At the very least, though, it appears that PRMT5-mediated methylation affects the efficiency with which snRNPs interact with specific splice sites in some clock transcripts.

PRMT5 also plays a role in other circadian systems. Examination of *prmt5* mutants in *Drosophila* revealed alternative splicing in clock output pathways rather than in the core oscillator, indicating evolutionary divergence between plants and animals [111,112]. Later studies in *Neurospora* showed PRMT5 is involved in the regulated splicing of the circadian clock gene *frequency* (*frq*) [113].

JMJD5 contains a jumonji-C domain that is often found in proteins with histone demethylase activity. In Arabidopsis *JMJD5* transcripts oscillate with an early evening phase, similar to *TOC1* [114]. A *jmjd5* mutant shortens the circadian period, and can be rescued by a mammalian *JMJD5* ortholog with validated histone demethylase activity, strongly suggesting a similar function in plants. *CCA1* and *LHY* transcripts are reduced in *jmjd5*, consistent with the short circadian period of the *cca1 lhy* mutant, but no further work has been reported concerning potential targets of this demethylase [114].

### 2.6. Phosphatidic Acid

Phosphatidic acid (PA) is a relatively new class of lipid mediators that plays roles in diverse cellular functions in plants, animals, and microorganisms [115]. PA target binding can regulate protein activity through recruitment, or by causing direct conformational changes [116]. An Arabidopsis transcription factor cDNA library screened for PA-interactors identified CCA1 and LHY [117]. PA association with LHY and CCA1 inhibited DNA binding, and increased PA levels lengthened period while reduce levels shortened period. Some PA species oscillate in abundance and perturbation of PA levels altered circadian outputs. These and other findings suggest a reciprocal regulation between the circadian system and PA levels. Given that cellular PA levels are stress responsive, PA may act as a metabolic connection between the circadian clock and biotic and abiotic stresses [117].

## 3. Post-Translational Mechanisms: Protein Partitioning and Movement

### 3.1. Nucleocytoplasmic Partitioning

Dynamic changes in nucleocytoplasmic partitioning of key clock proteins are another aspect of the post-translational regulation of the circadian system. Since the coordinated action of core clock proteins helps orchestrate the transcription of output genes in the nucleus, the nuclear translocation of these proteins is a pivotal regulatory point. The control of nuclear entry of key clock proteins in metazoans is often through phosphorylation or dimerization [118,119], and can also involve interaction with nuclear pore-related components and large cytosolic complexes (e.g., NRON) [120,121,122,123]. While nucleocytoplasmic trafficking has been well-studied in plants [124,125], little is known of its role in the circadian system.

In Arabidopsis, most of the key clock factors are nuclear localized [29,126,127,128,129,130,131], and one of the first studies to look at the trafficking of clock proteins investigated CCA1, which forms a heterodimer with LHY in the nucleus and functions as a transcription repressor. Time series analysis with CCA1 suggested that its nuclear import occurs very rapidly after translation, without a delay greater than the time resolution employed in the study [132].

The tripartite Evening Complex (EC), comprised of ELF3, ELF4 and LUX, are key transcriptional control elements of the plant circadian system that are expressed late in the day [71,72,133]. All three components are primarily nuclear-localized, with putative nuclear localization sequences (NLSs), but an interaction between ELF3 and ELF4 facilitates a stronger nuclear localization of ELF3 while the nuclear presence of LUX, the DNA-binding member of the complex, is not affected by the absence of either ELF3 or ELF4 [127,128,134]. These results are supported by single residue mutations in the ELF4-interacting middle domain of ELF3 which shifts its accumulation toward the cytosol [135,136], although whether a reduced interaction between ELF3 and ELF4 results from these mutations has not been directly tested. When ELF3 and ELF4 are co-expressed they preferentially form nuclear foci [134]. These foci may be points of EC complex positioning at chromatin binding sites, as ELF4 substantially increases the ELF3-LUX interaction, and subsequent DNA binding [137]. This notion is supported by recent work suggesting that ELF3 foci formation is temperature-dependent and is mediated by a prion-like domain present at the C-terminus. ELF4 can stabilize ELF3 chromatin presence, and partially restore the reduced binding of ELF3 to the target promoters at high temperatures [73]. The putative NLSs in both proteins suggest an importin-based nuclear entry, but how ELF4 accentuates ELF3 nuclear levels is unclear.

ELF4 also influences the sub-nuclear localization and activity of GI. The subcellular distribution of GI is important to its function [138], acting as a co-chaperone in the maturation of cytosolic ZTL [33] and as a transcriptional regulator in the nucleus [139,140]. In the nucleus, ELF4 promotes the localization of GI to nuclear bodies [141]. GI sub-nuclear speckles cycle with a peak at night under diurnal conditions. While sequestered within nuclear bodies by ELF4, GI presence at the *CONSTANS* promoter is diminished, resulting in later flowering [141], and ELF4 likely negatively regulates the overall transcriptional activity of GI in this way. Hence, in the context of ELF4-ELF3 nuclear bodies ELF4 appears to promote transcription through increased chromatin presence (in the context of the EC), whereas ELF4-GI nuclear bodies act to reduce GI-mediated transcription through sequestration from the chromatin.

In parallel, ZTL balances the cytoplasmic distribution of GI through effects on stability, as noted earlier [31], but also can sequester some portion of GI to the cytosol [142]. Ectopically expressed ZTL N-terminus containing either the LOV or LOV-F-box domain impairs the interaction between endogenous ZTL and GI through competitive binding to GI. Consistent with the finding that GI stabilizes ZTL [31], endogenous ZTL levels decline in the ZTL LOV-domain overexpressing plants. ZTL-LOV overexpression also elevates cytoplasmic GI levels resulting in late flowering due to a reduction in nuclear GI abundance [138,143]. These results suggest that a mutual stabilization of ZTL and GI occurs in the cytoplasm, and GI stabilization and cytoplasmic retention occurs naturally through a LOV domain mediated GI-ZTL interaction, with ZTL indirectly regulating GI nuclear pools by sequestering GI to the cytosol. This notion is supported by a recent study in which the reduced strength of GI-ZTL interactions in the Arabidopsis Cape Verde Islands ecotype, correlate with the low ZTL abundance [144]. The GI L^712^XXLXXL^718^ motif mediates GI-ZTL interaction and also determines GI nucleocytoplasmic partitioning.

ZTL facilitates TOC1 and PRR5 degradation in the cytoplasm [9,13], but PRR5 mitigates this turnover by boosting the nuclear import of TOC1 [8]. PRR5 interacts with TOC1 via their N-termini and enhances the nuclear abundance of the phosphorylated TOC1. A TOC1 N-terminal fragment localizes solely to the cytoplasm, but PRR5 expression strongly shifts TOC1 N-terminal fragment distribution into the nucleus, indicating that PRR5 promotes the nuclear entry of TOC1 instead of affecting protein stability or nuclear export. The temporal overlap in the expression of both proteins toward the early evening suggests that endogenous heterodimer formation is important in determining nuclear levels of both proteins.

Although these complex associations between clock-associated proteins suggest ways of fine-tuning nucleocytoplasmic partitioning, there are remaining questions of how the nuclear translocation of these proteins is achieved. Given the fact that many clock proteins have inherent NLS motifs, fundamental nuclear import/export pathways likely underlie their movement [145,146]. Differences in the temporal expression patterns of the proteins and their relative affinities for each other are likely among the many ways their basic trafficking mechanism is modulated.

### 3.2. Tissue-Specific Clocks and Intercellular/Interorgan Coupling

Early understanding of the plant clock was largely confined to studies on the whole organism. However, some early work with bean leaves showed different periods for leaf movement rhythms and stomatal rhythms [147], suggesting the existence of more than one type of circadian oscillator in plants. The advent of different tissue-specific promoters fused with luciferase reporters increased the repertoire of luminescent markers able to follow circadian rhythms in Arabidopsis. Work from the Millar lab reported different periods using *CAB-*, *phyB-* and *CHS-luciferase* reporters, and was the first to extensively document the likelihood of multiple clocks that are tissue-, cell- or organ-specific [148,149]. A recent report documenting shorter periods in older Arabidopsis leaves may be tied to changes in the clocks of specific leaf tissues or cells as they age [150].

**Figure 2:**
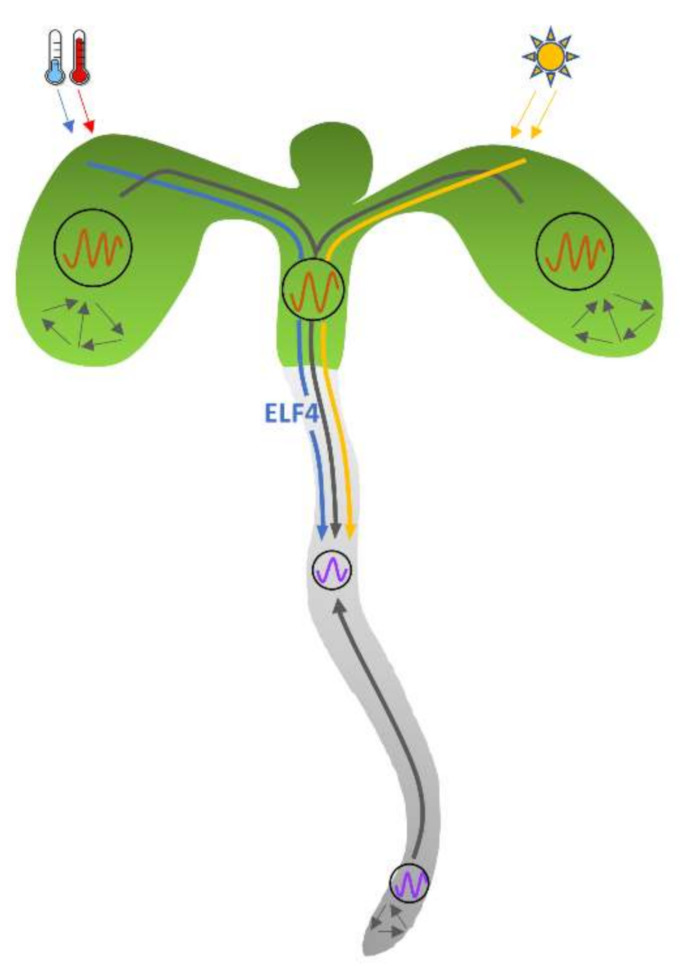
The coordination of the plant clock within and between tissues. Rhythms across the plant exhibit tissue specific phases and periods. Single cell imaging suggest local cell-to-cell coupling (indicated by short gray arrows pointing to each other) and long-distance coordination of clock by spatial waves of clock gene expression (indicated by long gray arrows). Tissue-grafting experiments show that shoots from wild-type plants restore the period in roots of arrhythmic clock mutants. ELF4 can move from shoots to roots to influence the root clock. Low temperature enhances ELF4 trafficking, which results in the lengthened circadian period, whereas high temperature inhibits ELF4 movement, shortening the period in roots (the blue arrow in Figure 2 indicates shoot-to-root movement of ELF4, which is enhanced by low temperature). Light piped from shoots has been also shown to entrain the root clock in seedlings (indicated by yellow arrow).

Recently, significant progress has been made in deciphering the molecular network of clock regulated synchronization of developmental and physiological processes. Work from multiple groups have revealed tissue-specific clocks with distinct rhythmic properties and which can reciprocally affect one another [151,152,153,154,155,156] (Figure 2). Direct tissue isolation coupled with global gene expression profiling indicate more robust and sustained rhythms in vasculature than mesophyll cells, and inverse gene expression profiles in vasculature compared to whole leaf and mesophyll [151]. A spatiotemporal luciferase complementation assay, driven by clock and tissue-specific promoters, helped to reconfirm the existence of divergent properties of circadian clock regulation in the vasculature [30,151]. Organ dissection and grafting experiments showed more robust and precise rhythms in the shoot apical meristem, in contrast to a longer period and dampened rhythms in the hypocotyl and root [157].

Interestingly, expression of CCA1 in vasculature perturbs the clock in mesophyll cells and further delays photoperiodic flowering by reducing *FT* expression, whereas CCA1 expression in epidermis, shoot apical meristem or hypocotyl/root has no effect on vasculature circadian rhythm and flowering, suggesting vasculature clock is dominant to other clocks and can regulate a whole plant physiological response [151,152]. However, clock function analyses in other tissues found cell elongation is regulated specifically by the epidermal clock, suggesting that unlike the centralized mammalian clock system, the plant clock is rather decentralized where each tissue specifically regulates individual physiological processes in response to environmental cues, such as photoperiod. The vascular-clock-dependent flowering response and the epidermal-clock-dependent cell elongation are both temperature-sensitive, indicating thermal signals can be also processed by tissue-specific clocks [152] (Figure 2).

The existence of tissue-specific clocks raises questions about local cell-to-cell and long-distance organ-to-organ communication. Coupling of central and peripheral clocks in mammalian system has been well-established and is achieved by the coordination of the hypothalamic suprachiasmatic nucleus (SCN), a central pacemaker in the brain [158,159]. By performing shoot excision and micrografting, Takahashi et al. (2015) found grafting of the shoot apexes of arrhythmic mutant plants onto a wild-type rootstock disrupts the rhythms in roots. In contrast, reciprocal grafting, in which wild-type shoot apices were grafted into arrhythmic rootstocks partially restores the rhythms in roots [157]. These results suggest that the shoot apical meristem can orchestrate, or at least influence, the clock in distal organs, similar to the mammalian suprachiasmatic nucleus [157].

Single-cell studies of rhythmicity luciferase-based clock reporters revealed robust but desynchronized oscillation in individual cells [154]. Using time-lapse imaging, two spatial waves of clock gene expression were observed in roots, one up from root tip and the other one down from hypocotyl junction, suggesting that the Arabidopsis clock has multiple coordination points [154], and a less hierarchical clock structure than that suggested from grafting experiments. Furthermore, rhythm analyses across entire seedlings demonstrated period and phase differences between organs, as the cotyledons and hypocotyl exhibited shorter periods and an earlier peak than the root, but oscillations in the root tip ran faster than the middle region of the root [160] (Figure 2). These observations are also qualitatively similar to the periods and phases previously observed in isolated organs [153,155,157], suggesting intra-organ heterogeneity in clock function.

Further examination at the sub-tissue level revealed spatial expression waves within and between organs both in constant and entrained conditions [160]. Sectioning seedlings at the hypocotyl junction and root tip does not substantially affect either the phase of the rhythms, period differences between tissues or the spatial gene expression waves, suggesting that rhythms are autonomous and the spatial waves that travel between them are not dependent on long-distance signals [160]. Further mathematical modeling together with the experimental results showed that the spatial waves are driven by the period differences between organs and local coupling [160]. By manipulation of environmental inputs, either via light or photosynthetic sugar, Greenwood et al. were able to modulate the waves in a predictable manner by locally altering clock periods. They proposed that the plant clock is set locally by tissue specific inputs but coordinated globally by spatial waves of gene expression [160]. However, other inputs, such as temperature cycles, light piped from the stem, or phloem mobile signals may also act to synchronize the root with the shoot [152,153,160,161].

The molecular bases of the interactions described above remain largely speculative. Clock coupling between cells and organs relies on mobile signals that can travel between cells and tissues. A number of signals, such as carbohydrates, nutrients, mRNA, and the transcription factor HY5, which are known to be mobile and capable of influencing clock, have been suggested as potential candidates for tissue-specific clock communication [162,163]. For example, sucrose has been shown to post-transcriptionally stabilize GI levels in the dark, dependent on ZTL [164]. Hence, sucrose transport from shoot to root could potentially influence period in the roots through adjustments in GI levels.

Chen et al. (2020) provided recent direct evidence that the small clock protein, ELF4, moves from shoots to roots and conveys thermal information between clocks in different tissues (Figure 2). Grafting ELF4-ox or wild-type shoots into *elf4-1* rootstocks restores rhythmicity in roots. Shoot injection of purified ELF4 protein and grafting of ELF4-GFP shoots both demonstrated ELF4 protein moves from shoots to roots and is capable to modulate the rhythms in roots. Additionally, blocking ELF4 movement by shoot excision alters circadian rhythms in roots, indicating shoot-to-root ELF4 movement can influence the root clock [165].

Analyses of temperature responses show that low temperature enhances ELF4 trafficking which results in slower-paced clock, whereas high temperature attenuates ELF4 movement from shoots to roots that leads to a faster root clock [165]. Further well-designed investigations are needed to demonstrate its role in clock coordination [160,165].

## 4. Perspective

While the transcription-translation feedback loop (TTFL) model of the circadian clock in eukarotes has prevailed for many years, evidence from multiple sources and organisms suggest cytosolic processes may contribute to sustaining robust circadian oscillations and even affect the circadian period [166,167,168]. Oscillations in cytosolic calcium in plant cells, for example, can connect to the TTFL from the Ca^2+-^dependent action of CALMODULIN-LIKE24 (CML24) acting through a TOC1-dependent signaling pathway to alter the levels of the clock-associated transcription factor *CHE* [169]. However, evidence for a purely cytosolic oscillator in eukaryotes that is solely sustained by changes in PTMs of clock proteins, similar hypo/hyperphosphorylation cycles of KaiC in the remarkable posttranslational oscillator of the cyanobacterium *Synechococcus elongatus* [170], is lacking. In eukaryotes the most extensively studied PTM, phosphorylation, is generally found to alter period and amplitude [63,171], but is not essential to sustaining the oscillator. Similarly, in plants phosphorylation likely acts as a modulator of the circadian system.

Of the multitude of possible protein modifications, many are associated with plant circadian factors. The increasing sensitivity of mass spectrometric and genome-wide proteomic techniques is refining our knowledge of when and where the additions and removals of these different moieties are occurring. However, interpretation of their functional meaning is becoming complicated by high resolution cell-specific and tissue-specific studies of the clock [156,172]. These reports are suggesting various permutations of the “core clock”, with some components possibly absent or expressed at different levels depending on the tissue or cell type. Most of the PTM studies described above invariably use plant extracts from whole seedlings, obscuring any potential differences between the tissues within the leaf, stem (hypocotyl) or roots. Unlike many metazoan systems where different tissues can be dissected out for individual harvest and study, similar work in plants can be exceedingly labor intensive, as well as difficult to obtain sufficient amounts for protein analysis. Possibly with the advent of single cell proteomics we will be able to obtain a better resolution of circadian heterogeneity in plants [173].
